# Influence of COVID-19 on the notification of drug-resistant pulmonary tuberculosis cases

**DOI:** 10.1186/s12879-023-08463-x

**Published:** 2023-07-28

**Authors:** Nathalia Halax Orfão, Rubia Laine de Paula Andrade, Antônio Ruffino-Netto, Leila Wiedmann Florentino da Silva, Tereza Cristina Scatena Villa, Marva Lynn Seifert, Adriana Zilly, Marcos Augusto Moraes Arcoverde, Ismael Hoare, Ricardo Izurieta, Reinaldo Antônio da Silva-Sobrinho

**Affiliations:** 1 Universidade Federal de Rondônia, Porto Velho, Brazil; 2 Universidade de São Paulo, São Paulo, Brazil; 3 Universidade Estadual do Oeste do Paraná, Foz do Iguaçu, Brazil; 4 University of California, San Diego, USA; 5 University of South Florida, Tampa, USA

**Keywords:** COVID-19, Tuberculosis, Drug resistance, Epidemiology

## Abstract

**Background:**

To analyze the influence of the COVID-19 pandemic on the process of diagnosis and monitoring of drug-resistant pulmonary tuberculosis (TB) cases reported in the state of Paraná, Brazil, from 2015 to 2020.

**Methods:**

Ecological study with quantitative approach. This study was based on diagnosed cases of pulmonary TB reported in the Notifiable Disease Information System in residents of Paraná; as well as through the number of confirmed cases of COVID-19 in the state epidemiological bulletin for the year 2020. The study data were analyzed using descriptive statistics.

**Results:**

It was found that, although the number of reported pulmonary TB cases (drug-resistant and general) increased between 2015 and 2019, there was a drop in notification in 2020, the first year of the COVID-19 pandemic. The notification of TB cases was also influenced monthly during the year according to the increase in the number of COVID-19 cases. For cases of drug-resistant pulmonary TB, the provision of diagnostic tests and Directly Observed Treatment decreased by more than half in 2020, especially when compared to 2019.

**Conclusions:**

In view of these findings, the influence of COVID-19 on the diagnosis and monitoring of drug-resistant and general pulmonary TB cases is evident, showing that the pandemic has compromised the advances of recent decades in achieving the goals established for its eradication by 2035.

## Background

Tuberculosis (TB) is recognized as a re-emerging and neglected public health problem, with the numbers remaining alarming in terms of incidence rates (127 cases/100,000 inhab.) and mortality (22 deaths/100,000 inhab.) [1], as well as weaknesses in delayed diagnosis and treatment, and therapeutic adherence, which increase cases of resistance [2].

The identification of drug resistance in TB occurs through the rapid molecular assay (RMA-TB) or sensitivity test (ST) and sequencing, and is considered critical for the institution of an effective treatment for the disease [1, 3]. However, the low request for and performance of tests to identify cases of resistance has historically been recurrent, contributing to underdiagnosis and, consequently, underreporting and lack of knowledge about the magnitude of the disease. Furthermore, the institution of inadequate treatment increases the challenges that involve the complexity of drug-resistant TB cases, leading to a worse prognosis of cases and an additional risk for the transmission of already resistant strains [1, 4–6].

The COVID-19 pandemic scenario changed the priority of health systems, which focused their efforts on controlling the disease, changing the organization of health services, funding and following programmatic goals [7]. This had an impact, for example, on reducing the number of cervical cancer prevention exams in Peru, on prenatal, dermatological and ophthalmological consultations in Paraguay and on the distribution of medications for the treatment of chronic conditions in Argentina [8, 9]. In Brazil, there was a 49% reduction in visits to Primary Health Care (PHC) units and a 25% reduction in consultations with specialists [10, 11].

Considering that, before the pandemic, about 3 to 4% of TB cases were resistant to antimicrobials and that 18 to 21% of these had received previous treatment for the disease, as well as the urgent need to restore access and care management to the people with TB [1, 12], this study aimed to analyze the influence of the COVID-19 pandemic on the process of diagnosis and monitoring of drug-resistant pulmonary TB cases reported in the state of Paraná, Brazil, from 2015 to 2020.

## Methods

This was an ecological study, with a quantitative approach, developed in the state of Paraná, Brazil, which had an estimated population of 11,516,840 inhabitants in 2020 and a territorial area of 199,298,982 km [2], with 399 municipalities and 22 Health Regions [13].

Regarding TB, in 2019, 2,357 new cases and 158 deaths were reported, which represent incidence and mortality coefficients of 20.6 and 1.38/100,000 inhabitants, respectively [14, 15].

The study population consisted of all cases of pulmonary TB diagnosed and reported in the Notifiable Diseases Information System (SINAN) of Paraná between 2015 and 2020. Drug-resistant cases were considered to be those with a termination status recorded as drug resistant TB (DR-TB) or a resistant result in the sensitivity test (ST) or detectable rifampicin-resistant in the rapid molecular test (RMT-TB). People residing in other states were excluded.

The data search was carried out in the SINAN considering the following variables: year of diagnosis, diagnostic tests (sputum smear and sputum culture, X-ray and HIV), Directly Observed Treatment (DOT), and termination status. In a complementary way, the number of confirmed cases of COVID-19 was obtained from the epidemiological bulletin of Paraná for the year 2020.

Data were stored and analyzed using descriptive statistics. To verify the correlation between the cases (TB/resistant TB and COVID-19), the Pearson correlation coefficient (*r*) was calculated using the Microsoft Excel Program. For a better visualization of the findings, they were presented by means of a graphic representation.

This study used a database with aggregated information, without the possibility of identifying the subjects with access information and public domain, which negated the need for approval of this study by the Ethics Committee for Research with Human Subjects and the consent from participants, in accordance with Resolution No. 510 of 07 April 2016, of the National Health Council. All methods are carried out in accordance with relevant guidelines and regulation.

## Results

**Fig. 1:**
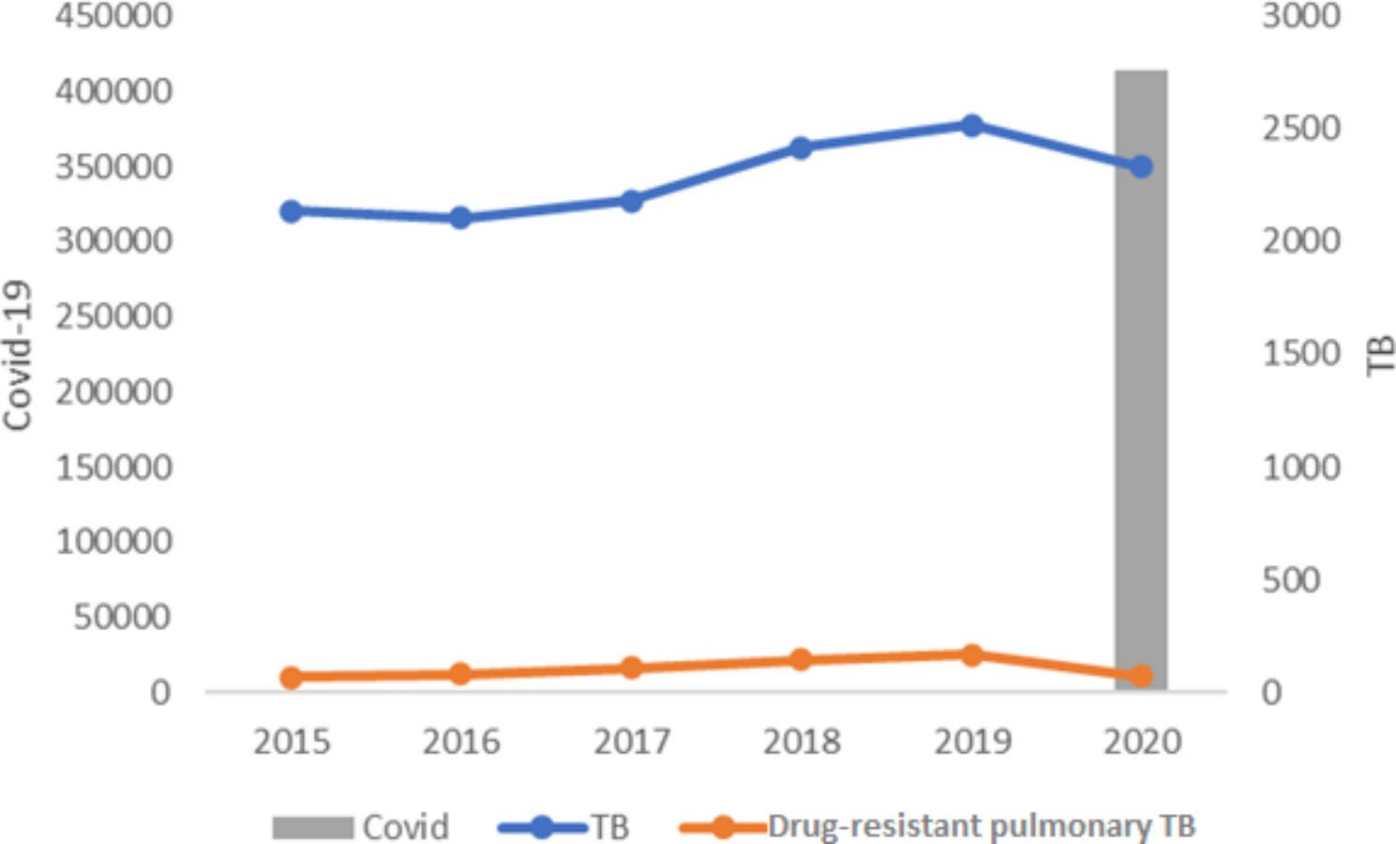
Quantitative distribution of drug-resistant pulmonary TB and pulmonary TB cases reported in the SINAN and confirmed cases of COVID-19 according to the epidemiological bulletin for the state of Paraná, Brazil, 2015 to 2020* * The pandemic started in the year 2020.

In the period from 2015 to 2020, 16,719 cases of TB were reported in the SINAN, of which 2,973 were excluded for having the extrapulmonary and mixed clinical form, and 65 due to residing in another state. Although the notification of the number of drug-resistant pulmonary TB cases increased between 2015 and 2019, there was a drop in 2020, the year of the COVID-19 pandemic evaluated, in which the trend followed the annual number of pulmonary TB cases in general (Fig. 1).

**Fig. 2:**
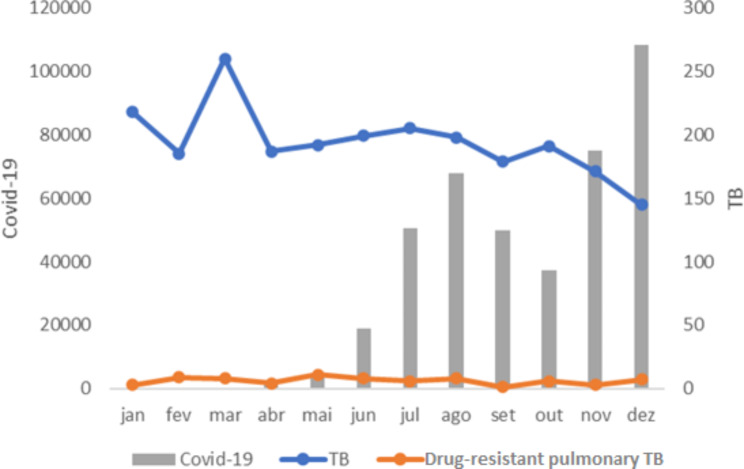
Quantitative distribution of drug-resistant pulmonary TB and pulmonary TB cases reported in the SINAN and confirmed cases of COVID-19 according to the epidemiological bulletin in the state of Paraná, Brazil, 2020

In 2020, 413,412 cases of COVID-19 were reported. The increase in the number of confirmed cases for the disease in some months of the year led to a decrease in the notification of drug-resistant pulmonary TB cases. This repercussion was more evident in relation to pulmonary TB in general (Fig. 2).

**Fig. 3:**
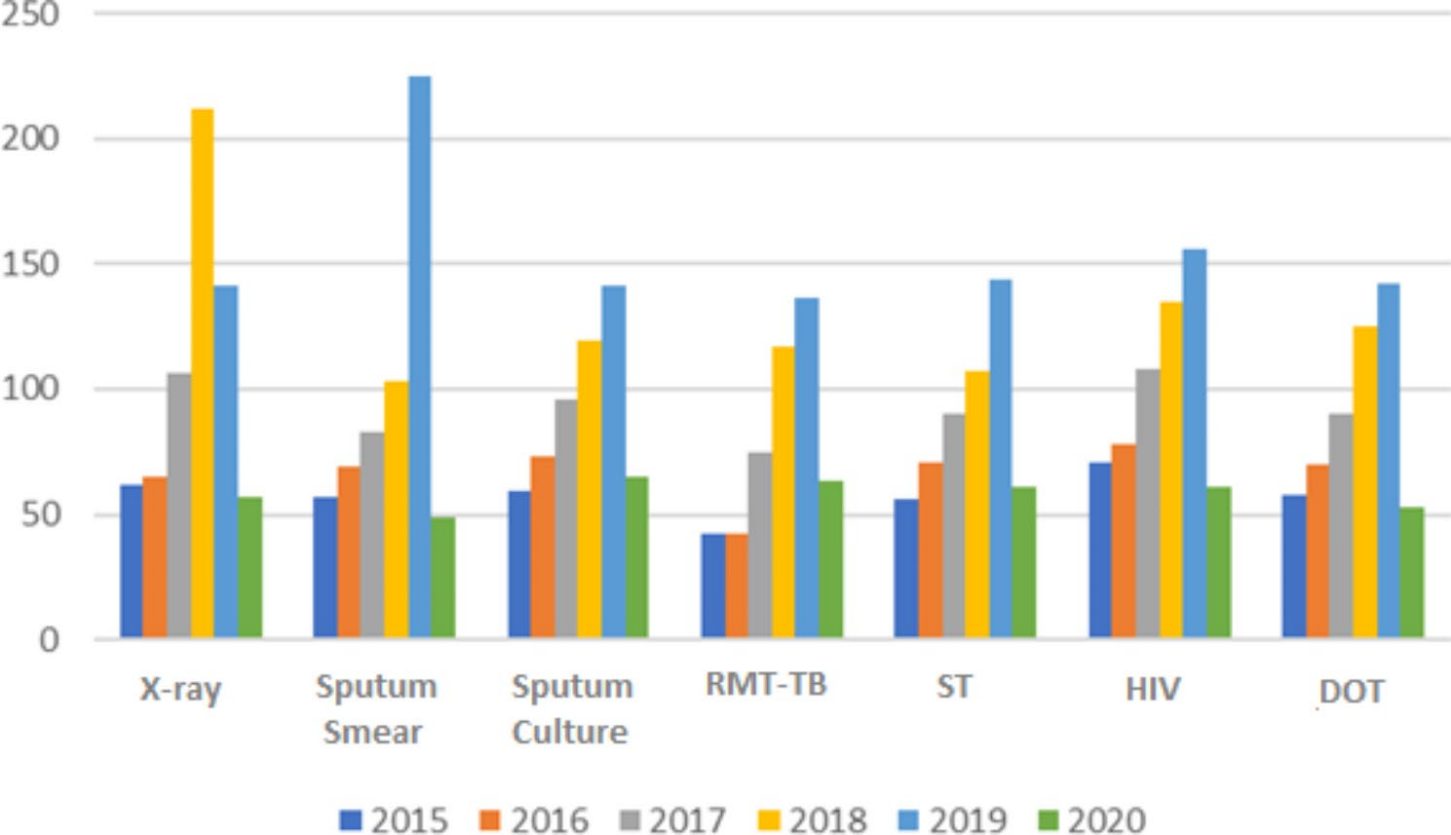
Distribution of drug-resistant pulmonary TB cases reported in the SINAN, according to diagnostic tests and performance of DOT, in the state of Paraná, Brazil, 2015 to 2020

Legend: RMT-TB: Rapid Molecular Test for TB; ST: Sensitivity test; DOT: Directly Observed Treatment; HIV: Human Immunodeficiency Virus.

For cases of drug-resistant pulmonary TB, the provision of diagnostic tests, HIV exams and Directly Observed Treatment (DOT) decreased by more than half in 2020, especially when compared to 2019 (Fig. 3).

**Fig. 4:**
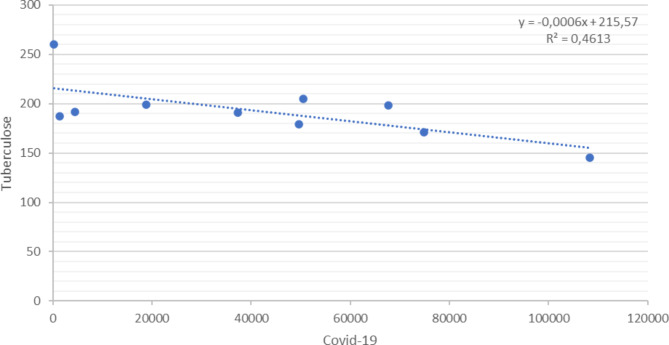
Pearson’s Correlation Test between the number of pulmonary TB cases reported in the SINAN and confirmed cases of COVID-19 according to the epidemiological bulletin in the state of Paraná, Brazil, 2020

**Fig. 5:**
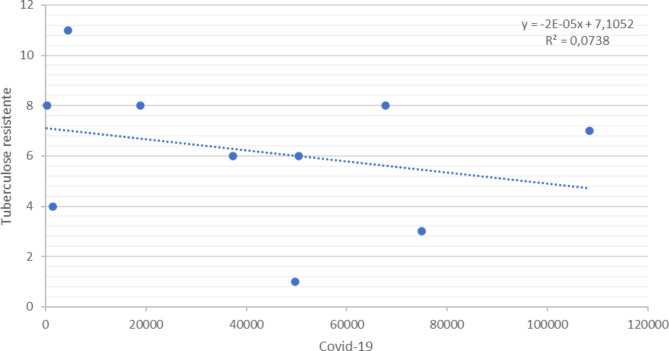
Pearson’s Correlation Test between the number of drug resistant pulmonary TB cases reported in the SINAN and confirmed cases of COVID-19 according to the epidemiological bulletin in the state of Paraná, Brazil, 2020

Figures 4 and 5 present Pearson’s linear correlation coefficient, between TB and COVID-19 cases (*r* = − .6792) and resistant TB and COVID-19 (*r* = − .2716), in 2020, which showed negative correlations.

## Discussion

Considering the findings, an initial reflection on the decrease in the number of reported cases of TB and drug-resistant pulmonary TB becomes necessary, since health systems were overloaded with the COVID-19 pandemic, limiting the provision and realization of TB diagnostic tests. This was due to the need to reorganize care and to prioritize the fight against the pandemic by managers and healthcare providers [16–19], whose decision-making was often carried out based on insufficient and outdated evidence [20].

At the same time, it is necessary to consider that many people postponed and/or stopped using health services and intentionally monitoring their health conditions for fear of getting sick with COVID-19, reflected as a subjective perception that contact with the care network would be an exposure or risk factor for SARS-CoV-2 infection [20].

As a result, not only Paraná, but the entire country showed a reduction in the number of notifications of active and latent TB cases, and in the performance of RMT-TB and STs, contributing to the underdiagnosis or late diagnosis of the disease. In addition, treatment interruptions were identified, which could increase the risk of unfavorable outcomes and the development of antimicrobial resistance [1, 21, 22]. It was also found that COVID-19 contributed to a 15% decrease in cases receiving treatment for drug-resistant TB [1].

These points are also a reflection of the directing of financial resources and the attention of managers, the population and the media toward a response to the pandemic, as well as the overload and mental suffering of healthcare providers who adopted coping strategies and adaptive behaviors that were not consistent with the care needs for diseases other than COVID-19 [21, 23, 24].

As important risk factors for infection and evolution of TB, the economic and social impact of the pandemic should also be highlighted, as this resulted in increased poverty and worse nutritional and housing conditions, with the agglomeration of family members in restricted spaces [21, 24]. Accordingly, the need for social protection and support for the patient and family should be reinforced, as a strategy to mitigate the impact of the pandemic on TB control actions, considering the vulnerabilities and social determinants of health that require the effectiveness of health systems to comply with the principles of equity, universality and integrality [21, 24–26]. Considering the economic and social impacts of the pandemic and responding to these issues could help to stop the risk of transmission of drug-resistant TB, which is 8% higher among household contacts when compared to sensitive cases [26–29].

In a complementary way, it is necessary that health services redirect their efforts to the implementation and effectiveness of the line of care for the person with TB and to overcoming the fragmentation of care and management of patient-centered care, having PHC as a point of preferential attendance for the care and coordination of cases [22]. Therefore, the need for communication and coordination between healthcare providers (horizontal integration) and health services (vertical integration) is emphasized, considering the technological complexity, for the continuity of care and co-management of TB cases [3, 25].

It is essential to reflect on the organization of services that make up the Health Care Network, as well as on the role of PHC in meeting the requirements and needs in health territories, in addition to the work process, in which the prioritization, when necessary, for the provision of DOT, for example, makes it possible to contribute to overcoming the challenges and barriers of access to TB control.

Studies indicate more underreporting of diagnostic tests and chest X-rays among patients with TB resistant to antimicrobials [30] or suggestive of TB [26], as well as not performing DOT [12, 29, 30], even in periods outside the context of the pandemic. Such weaknesses lead to a lack of knowledge about the real magnitude of the problem, and also have an impact on the planning and implementation of health surveillance actions and the fight against TB, which were enhanced in the context of the pandemic with the low number of requests for diagnostic tests.

Acting in the prevention-diagnosis-treatment triad becomes essential, with the development of biosafety measures, including the training of healthcare providers, psychosocial support, incentives to maintain the quality of TB services, integration of Community Health Agents in the actions of surveillance and investigation of contacts, as well as public participation and social actions for coping with and controlling TB [19, 21, 24].

In the diagnosis, the management of TB care during COVID-19 must be prioritized, with expansion of simultaneous screening for both diseases and continuous provision of exams and the laboratory network for support. In the treatment, emphasis is on the supply of drugs, establishment, implementation and (re)knowledge of the flow of referrals and counter-referrals, particularly for the vulnerable population, as well as the use of technological tools to monitor adherence to treatment and, consequently, allow the integrality and longitudinality of care through digital platforms, as is possible with DOT through video observation [1, 19, 21, 24, 31–33].

The centralization of health services for the care of COVID-19 contributed to its priority over other diseases, such as TB, given the shortage of human, material and financial resources, postponing other requirements that were not related to the pandemic.

The response to combating COVID-19 with the managerial capacity to act beyond the clinical aspects, with access to information technology, projection of cases and beds, and hiring of professionals could be maintained and directed toward TB control to ensure continuity and comprehensiveness of care for people affected by the disease, which could also be extended to people with other health conditions.

Finally, it should be noted that COVID-19 also affected spaces for debates, awareness-raising and discussions of research developed on TB, BCG vaccination coverage and the treatment of latent infection [1, 21], requiring the continuity of operational scientific research to support public policies for coping with the disease.

As a limitation of this study, weaknesses in the recording of data/information can be highlighted, with incompleteness possibly contributing to and interfering with the epidemiological reality of resistant pulmonary TB.

## Conclusion

Considering these findings, the influence of COVID-19 on the incidence of reported cases of pulmonary TB (in general and drug-resistant) was verified. This fact seems to characterize TB as a disease marginalized for public health and affected by the COVID-19 pandemic, compromising the diagnosis of TB (with a decrease in the supply of tests), the notification of cases, the performance of DOT and the governmental budget destinated for tuberculosis in the period, with setbacks that compromise advances achieved in recent decades and the goals set for the eradication of TB by 2035.

The challenges in monitoring TB cases during the COVID-19 pandemic may have contributed to the increase in antimicrobial resistance, even if these cases have not yet been diagnosed and/or reported by health services. Tuberculosis has presented itself as an unprecedented pandemic for decades and together with COVID-19 constitutes a syndemic. Accordingly, it is necessary to rethink the triad for TB (prevention-diagnosis-treatment), possibly including funding, in order to strengthen patient- and community-centered care and treatment, as this is present even during COVID-19 times.

## Data Availability

The datasets used and/or analysed during the current study are available from the corresponding author on reasonable request.
